# Vitamin D Status during Pregnancy: A Longitudinal Study in Swedish Women from Early Pregnancy to Seven Months Postpartum

**DOI:** 10.1371/journal.pone.0150385

**Published:** 2016-03-03

**Authors:** Anette Lundqvist, Herbert Sandström, Hans Stenlund, Ingegerd Johansson, Johan Hultdin

**Affiliations:** 1 Department of Public Health and Clinical Medicine, Family Medicine, Umeå University, Umeå, Sweden; 2 Department of Public Health and Clinical Medicine, Epidemiology and Global Health, Umeå University, Umeå, Sweden; 3 Department of Odontology, Cariology, Umeå University, Umeå, Sweden; 4 Department of Medical Biosciences, Clinical Chemistry, Umeå University, Umeå, Sweden; University of Alabama at Birmingham, UNITED STATES

## Abstract

Low vitamin D levels during pregnancy may have negative consequences for the health of both the mother and child. Cross-sectional studies in childbearing women suggest that vitamin D levels are low during pregnancy, but few studies have followed the same women during pregnancy and postpartum. The aims of this study were to longitudinally assess vitamin D status during pregnancy and postpartum and identify the factors associated with vitamin D status in pregnant women in northern Sweden. Between September 2006 and March 2009, 184 women were consecutively recruited at five antenatal primary care clinics. Blood was sampled, and dietary intake was estimated using a food frequency questionnaire with 66 food items/food aggregates and questions on the intake of vitamin supplements at gestational weeks 12, 21, and 35, as well as at 12 and 29 weeks after birth. Plasma 25(OH) vitamin D levels were analyzed using liquid chromatography tandem-mass spectrometry. At least one-third of the women had 25(OH) vitamin D levels <50 nmol/L on at least one sampling occasion. Plasma levels increased slightly over the gestation period and peaked in late pregnancy. The levels reverted to the baseline levels after birth. Multivariate analysis showed that gestational and postpartum week, season, dietary intake of vitamin D, and vitamin supplementation were significantly related to plasma levels. There was also an influence of season on the longitudinal concentration patterns. In conclusion, more than one-third of the women studied had low 25(OH) vitamin D levels, and gestational and postpartum week was related to 25(OH) vitamin D levels after adjustment for season and vitamin D intake.

## Introduction

Vitamin D receptors are present in cells, organs, and tissues, including the placenta-decidua tissue. Vitamin D is essential for the regulation of calcium and phosphorus homeostasis and plays a role in various biological actions, such as cell proliferation and differentiation, in many target tissues [1,2]. During pregnancy and lactation, the need for both calcium and vitamin D is increased due to fetal and infant development, particularly that of mineralized structures. To meet the increased need, 1, 25(OH)_2_D synthesis increases [3] together with alterations in hormones involved in calcium and vitamin D metabolism, e.g., prolactin, placental lactogen, calcitonin, and estrogen [3]. Low vitamin D levels have been associated with adverse birth outcomes and complications, such as gestational diabetes mellitus, preeclampsia, small-for-gestational age, and preterm birth [4].

Vitamin D is a fat-soluble prohormone that occurs in two forms: cholecalciferol (D_3_) and ergocalciferol (D_2_). Both forms are converted to calcidiol [25-(OH)D] in the liver. Vitamin D is further metabolized in the kidneys and other tissues by the enzyme 1-α-hydroxylase into the active steroid hormone calcitriol [1,25 (OH)_2_D]. Calcitriol synthesis is influenced by levels of calcium, phosphate, and parathyroid hormone, and calcitriol operates in tandem with parathyroid hormone to maintain calcium and phosphorus homeostasis and support bone mineralization and skeletal growth by regulating the absorption and excretion of calcium and phosphate in the intestine and kidneys [5,6]. Vitamin D status is estimated by measuring the 25(OH) D levels in serum or plasma [7]. Vitamin D insufficiency has been defined as serum vitamin D levels of <50 nmol/L [8,9].

Cholecalciferol (vitamin D_3_) is produced from 7-dehydrocholesterol in the skin under the influence of sunlight (ultraviolet B radiation; wavelengths 290–315 nm) [6]. The synthesis of vitamin D in the skin, which depends on adequate exposure to ultraviolet sunlight, is affected by skin pigmentation levels, age, obesity, clothing, use of sunscreen products, latitude, season, time of day, the ozone layer, and air pollution [10]. In northern Sweden (latitude 63.8°N), sun exposure is sufficient for vitamin D_3_ synthesis between 15 April and 15 September, whereas it is too low during the 7 winter months [11]. Thus, vitamin D intake from sources such as fatty fish, eggs, vitamin-enriched dairy products, and vitamin D supplements is important to ensure adequate levels during the winter [12]. At birth, infants have a store of vitamin D; however, as the vitamin D content in human milk is low, the store is depleted at approximately 8 weeks of age [3]. To balance the depleted vitamin D levels, infants in Sweden are recommended to receive 10 μg/day of vitamin D from 1–2 weeks to 2 years of age and even longer in some other countries [12].

Several studies have reported low levels of vitamin D in pregnant women. For example, Milman et al [13] found that low vitamin D status was common in Danish pregnant and lactating women with a low vitamin D intake (2.4 μg/day). Brembeck et al [14] reported that 85% of pregnant women in mid-Sweden (latitude 57–58°N) had plasma levels of <50 nmol/L during the winter. Similar findings have been reported in Belgium [15]. In contrast, a cross-sectional screening of vitamin D status in non-pregnant women living in a sub-Arctic part of northern Sweden (latitudes 63.8°N to 65.6°N) revealed that 82.7% had adequate levels of vitamin D (≥50 nmol/L plasma) based on samples collected between January and April [16]. In this study, the levels were lower in women aged 25–34 years (n = 130), with 22.3% having insufficient levels. We previously estimated that the mean dietary intake of vitamin D was as low as approximately 6.5 μg/day in both non-pregnant and pregnant women in the same geographic area [17], compared with the recommended intake of 10 μg/day [12]. These seemingly conflicting results on vitamin D status in Swedish women of childbearing age, along with the fact that the vitamin D time course during pregnancy and postpartum has not been studied longitudinally in women living in the sub-arctic region of Sweden, need to be addressed. Such information is important in maternal counseling as the conditions associated with health or disease susceptibility of a child is set by the environment in the uterus [18].

In this study, we aimed to longitudinally assess vitamin D status during pregnancy and postpartum and identify the factors associated with vitamin D status, including intake and seasonal variations, in pregnant women in northern Sweden (latitude 63.8°N).

## Subjects and Methods

### Ethics Statement

This study was approved by the Regional Ethical Review Board at Umeå University, Sweden (Dnr 04–171M). The study was conducted according to the principles established in the Declaration of Helsinki. Written informed consent was obtained from all participants. The antenatal clinics involved are all part of Västerbotten County Council and under the authority by this Ethical Review Board.

### Participants

**Fig 1:**
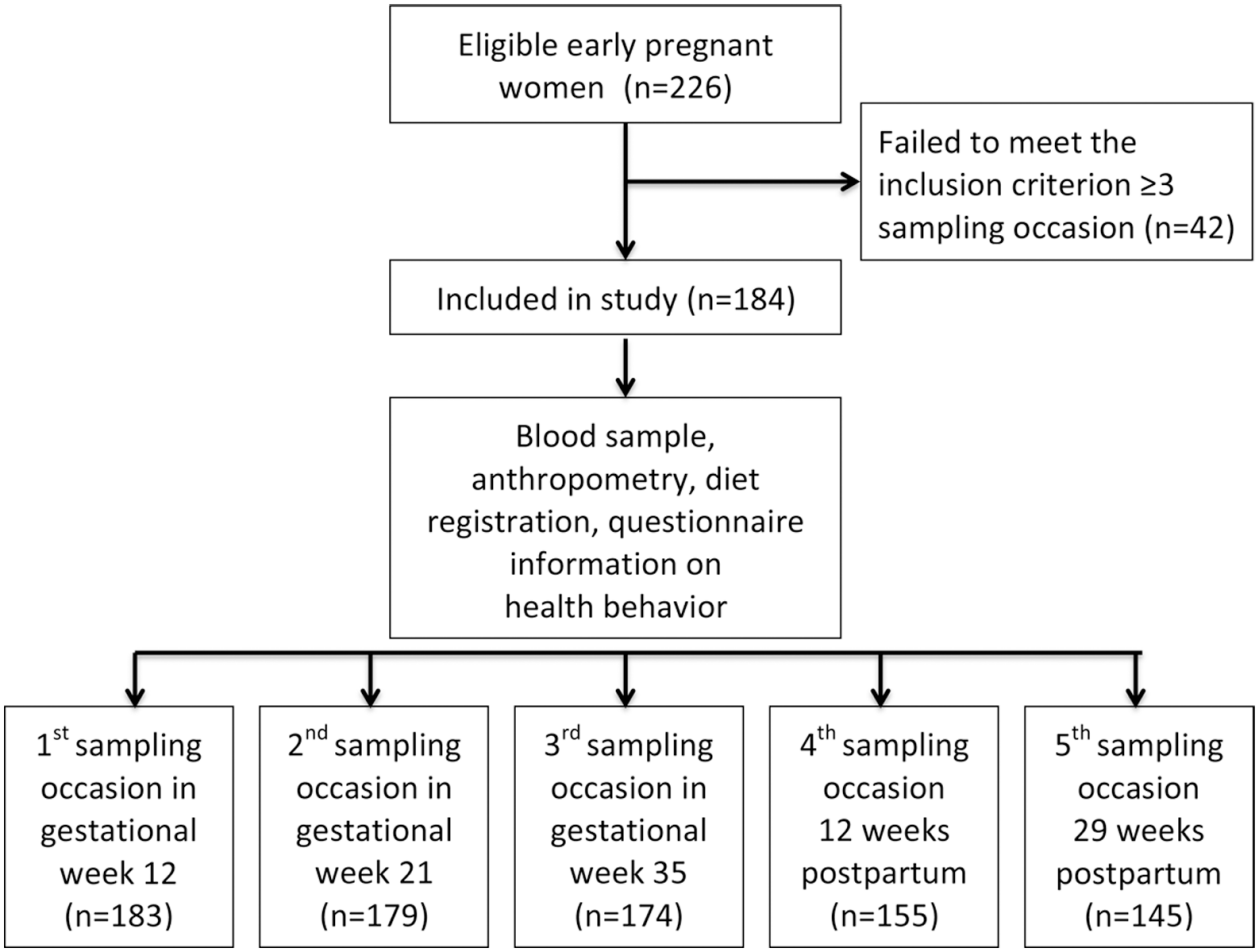
Flow diagram of the study population.

The present study is based on a cohort of 226 women followed from early pregnancy to postpartum [17]. The recruitment size of the cohort was chosen on the basis of a pre-study statistical power calculation based on t-test, using Sample Power 2 (SPSS, Chicago, IL). We used data from a study where 39 pregnant women were followed from first to third trimester [18], reporting changes for vitamin B12, folate, and homocysteine in plasma, with percentage changes 4.4, 8.6 and 4.3 respectively. Two-tailed significance test at alpha level 0.05 yielded a statistical power of 80% at sample sizes 176, 202 and 176 respectively. Women were consecutively recruited at five antenatal clinics in primary care in Umeå, Sweden (latitude 63.8° N), between September 2006 and March 2009. On their first visit to the antenatal clinics during the recruitment period, women were offered information on the study by midwives. Those who agreed were provided verbal and written information and invited to participate. Signed consent was obtained at the first visit to the antenatal clinic and at each subsequent blood sampling occasion. The exclusion criteria were as follows: major medical conditions, being unable to attend the antenatal wellness program, and insufficient competence in the Swedish language. A minimum blood sampling at three different visits was set as the criterion for inclusion in the present study. A total of 126 women participated on all five sampling occasions, 40 women on four and 18 women on three sampling occasions, resulting in a final cohort of 184 women. A flow diagram of the study is shown in Fig 1.

### Questionnaire

A thorough questionnaire on food intake and health behavior was administered at gestational weeks 12, 21, and 35, as well as at 12 and 29 weeks after birth (sampling occasions 1–5). The questionnaire, including questions regarding dietary intake, calculation of total energy, vitamin D intake, and other measurements, has been described previously [17].

Season was defined according to Engelsen [11] and linked to the current latitude of Umeå (63.8°N) to estimate the vitamin D level derived from sunlight. Season was dichotomized into “winter” (16 September–14 April) and “summer” (15 April–15 September). Age was classified as “≤34 years” or “≥35” years. Parity was either “nulliparous” or “multiparous.” Education was dichotomized into having a “university education” or “lower than university education.” Intake of multivitamins was reported as “Yes” or “No” for intake during the latest year and/or previous 2 weeks. Intake of vitamin D represents the estimated intake (*i*) through diet and (*ii*) with the addition of reported multivitamin intake. The most common content of over-the-counter preparations (7.5 μg vitamin D) sold to women during the study period was used to calculate total intake as described previously [17]. The total energy intakes were estimated, and the feasibility was judged in relation to the basal metabolic rate as described previously [17]. The body weight (light clothing) and height (no shoes) were measured, and body mass index was calculated and expressed as kg/m^2^ at each sampling occasion.

### Blood sampling and vitamin D analysis

Venous blood samples were collected into evacuated test tubes with EDTA. The samples were collected in the morning after an overnight fast and at the appointments when the information in the questionnaire was collected. Blood was centrifuged at 1500×*g* for 15 min, and the plasma was aliquoted and stored at −80°C until analysis. Liquid chromatography-tandem mass spectrometry (LC-MS/MS) was used at our laboratory to determine plasma levels of cholecalciferol (D_3_) and ergocalciferol (D_2_) using the MSMS Vitamin D Kit, 3075–0010 (Perkin Elmer, Waltham, MA, USA). The total 25(OH)D levels were calculated as 25(OH)D_2_ + 25(OH)D_3_. The total coefficients of variation for 25(OH)D_3_ were 13.9%, 11.4%, and 11.1% at levels of 21.7, 90.0, and 181.8 nmol/L, respectively, in this study. The corresponding values of 25(OH)D_2_ were 14.3%, 13.4%, and 9.5% at levels of 28.3, 119.4, and 248.0 nmol/L, respectively. The analytical reliability was ensured by external controls from DEQAS (www.deqas.org) with values assigned by the NIST Reference Measurement Procedure.

### Statistical analysis

Data are expressed as the means ± standard deviations at each sampling occasion. The distribution was found to be acceptably normal for all continuous variables. The mean values of vitamin D levels at each sampling occasion for different factors, e.g., age, parity, education, season, and multivitamin supplementation during the previous year and/or 14 days, were compared using Student’s *t*-test. The BMI associations were assessed using Spearman correlation coefficients.

A linear mixed model with an unstructured covariance matrix was used to analyze the relationship between 25(OH)D plasma levels and gestational week, squared gestational week, age, parity, education, BMI, season, total energy, vitamin D intake from food, and multivitamin supplementation during the previous year and/or 14 days, and the measurement occasion was measured as weeks from conception. Each factor was first tested in a univariate analysis. Then, factors that were statistically significant or considered relevant were included in the multivariate mixed model. Because the relationship between the 25(OH)D plasma levels and sampling occasion was nonlinear, the multivariate model included a squared term of gestational week. To illustrate longitudinal patterns, we plotted individual changes, dividing the women by month of first blood sampling occasion.

P < 0.05 was considered statistically significant. IBM SPSS Statistics version 22 (IBM Corporation, New York, NY, USA) was used for the data analyses.

## Results

The baseline characteristics of the 184 included women are shown in Table 1. There were no differences in the social parameters between the women who provided at least 3 blood samples and those who provided ≤2 blood samples.

**Table 1:** Baseline characteristics of the pregnant women. Data are presented as the means and standard deviation (SD) or proportions (%).

Characteristics (n = 184)	
Age (21–42 years) (mean (SD))	31 (4.4)
Ethnicity^1^ (%)	93.5
Married/cohabitant (%)	98.7
Education (% with university)	59.4
Nullipara/multipara (%)	62.2/37.8
Singleton/twin pregnancy (%)	97.8/2.2
Gestational length (weeks) (mean (SD))	39.4 (1.7)
Starting BMI (kg/m^2^) (mean (SD))	24.1 (3.8)
Reported total energy (kcal/day) (mean (SD))^2^	1,629 (476)

^1^Sweden as a country of origin.

^2^ Energy intake was increased by 25% to adjust for underreporting due to the short the FFQ as described previously version [17].

The plasma 25(OH)D levels at various stages of pregnancy and 12 and 29 weeks after birth are shown in Table 2. The 25(OH)D levels increased continuously from 55.2 nmol/L in early pregnancy to the highest level of 64.6 nmol/L in late pregnancy. After birth, the levels reverted to the values observed at the earliest pregnancy stage. The proportions of women classified into the two lowest 25(OH)D categories (plasma levels of <25 and 25–49.9 nmol/L) were virtually constant on all sampling occasions, whereas the proportions classified into the two highest 25(OH)D categories (plasma levels of 50–75 and >75 nmol/L) reflected the pattern observed for the mean concentration (Table 2).

**Table 2:** Plasma concentrations of 25(OH)D in women during pregnancy and after birth. Data are presented as the means and standard deviations (SD) or proportions (%) according to 25(OH)D category.

Sampling occasion	1	2	3	4	5
	(n = 183)	(n = 179)	(n = 174)	(n = 155)	(n = 145)
Gestational week/ Weeks after birth	12	21	35	12	29
25(OH)D nmol/L (mean (SD))	55.2 (17.5)	60.2 (22.5)	64.6 (27.3)	54.2 (16.4)	53.5 (16.6)
Proportion in 25(OH)D categories (%)					
<25 nmol/L	2.7	3.4	4.6	2.5	4.1
25–50 nmol/L	35.0	34.6	28.7	35.5	37.9
50–75 nmol/L	53.0	38.5	34.5	52.3	47.6
>75 nmol/L	9.3	23.5	32.2	9.7	10.3

The plasma 25(OH)D levels on the five sampling occasions and a list of factors potentially related to vitamin D status are shown in Table 3. The 25(OH)D levels did not differ significantly between the sampling occasions according to education, age, parity, and multivitamin intake in the previous year. However, the reported use of multivitamins during the previous 14-day period was significantly related to plasma 25(OH)D levels on sampling occasions 1, 4, and 5 but not 2 and 3. Season was significantly related to the 25(OH)D levels (*p* < 0.001), except on sampling occasion 4, i.e., 12 weeks after birth (*p* = 0.093). The plasma 25(OH)D levels were higher in samples collected during the summer. Spearman’s p (rho) showed a negative correlation coefficient -.106 (p = 0.159), -.080 (p = 0.292), -.150 p = (0.085) at sampling occasions one, two and four respectively indicated a nonsignificant relationship between body weight relative to height and 25(OH)D levels. At sampling occasions three and five a weak relationship with a negative correlation coefficients -.199 (p = 0.010) and -.226 (p = 0.015) was found.

**Table 3:** Plasma concentrations of vitamin D during pregnancy and after birth. Data are presented in mean and standard deviation (SD).

Sampling occasion	1		2		3		4		5	
	(n = 183)	*P*	(n = 179)	*p*	(n = 174)	*p*	(n = 155)	*p*	(n = 145)	*p*
Gestational week/weeks after birth	12		21		35		12		29	
Concentration of 25(OH) D (nmol/L) (mean (SD))										
Both seasons together	55.2 (17.5)		60.2 (22.5)		64.6 (27.3)		54.2 (16.4)		53.5 (16.6)	
Season^1^ (winter)	51.4 (16.9)		50.2 (17.2)		57.4 (25.4)		52.6 (17.4)		49.4 (16.8)	
Season^1^ (summer)	62.8 (6.2)	<0.001	69.6 (23.0)	<0.001	73.1 (27.1)	<0.001	57.2 (13.8)	0.093	59.0 (14.8)	<0.001
Multipara	53.6 (16.2)	0.559	55.9 (21.8)	0.048	61.8 (27.4)	0.293	54.3 (17.5)	0.950	51.0 (15.5)	
Nullipara	56.1 (18.2)		62.7 (22.6)		66.3 (27.2)		54.1 (15.8)		55.0 (17.1)	
Multivitamin supplement latest year (no)^2^	54.1 (19.1)		60.0 (23.3)		61.6 (27.7)		52.8 (16.1)		52.7 (16.0)	
Multivitamin supplement latest year (yes)^2^	56.6 (15.2)	0.347	60.4 (21.7)	0.892	67.9 (26.7)	0.129	55.9 (16.7)	0.244	54.5 (17.2)	0.515
Multivitamin supplement latest 14 days (no)^2^	52.9 (19.1)		58.7 (22.9)		63.8 (29.4)		52.8 (16.4)		51.9 (16.4)	
Multivitamin supplement latest 14 days (yes)^2^	58.4 (14.2)	0.027	62.9 (21.7)	0.222	66.0 (23.7)	0.591	59.5 (15.5)	0.040	61.0 (15.2)	0.010
Education (< university)	55.9 (17.1)		61.9 (22.0)		63.9 (26.5)		54.4 (17.0)		53.3 (15.9)	
Education (university)	54.7 (17.8)	0.660	59.0 (22.8)	0.395	65.1 (27.9)	0.765	54.0 (16.0)	0.892	53.7 (17.0)	0.892
Age (≤34 years)	55.5 (17.8)		59.9 (22.4)		63.8 (27.8)		54.7 (16.6)		53.0 (16.9)	
Age (≥ 35 years)	53.5 (16.0)	0.559	61.7 (23.3)	0.680	68.7 (24.8)	0.367	51.8 (15.7)	0.370	55.3 (15.4)	0.472
Dietary intake of vitamin D (μg/day)^3^	5.1 (1.9)		5.0 (2.0)		4.9 (1.8)		5.4 (2.1)		5.3 (2.4)	
Vitamin D with supplement latest year μg/d)^3^	12.6 (1.8)		12.4 (1.9)		12.4 (1.7)		13.2 (2.0)		13.0 (2.4)	
Vitamin D with supplement latest 14 days (μg/d)^3^	12.5 (1.7)		12.5 (1.9)		12.6 (1.6)		13.3 (1.9)		13.7 (3.1)	

p = Differences between groups within each time point (column) are tested with Student’s *t*-test.

^1^ Summer; April 15 –September 15, Winter; September 16 –April 14 [11].

^2^ Mean values represent estimated nutrient intake with addition from supplements in those who reported intake of multivitamin the latest year or 14 days. Addition has been done with the most common content in over-the-counter sold supplement targeting women at the time of blood sampling measurement, *i*.*e*. vitamin D 7.5 μg.

^3^ Nutrient intake increased by 25% to adjust for underreporting resulting from shortening of the FFQ by 25% from the original validated version [17].

The estimated intake of vitamin D from food was virtually similar on all occasions (mean intakes ranging between 4.9 and 5.4 μg/day; Table 3). Similarly, the estimated intake including the addition of vitamin D from supplements, which was nearly 2.5-fold greater than the intake from food, did not vary over the study period (Table 3).

The final multivariate mixed model in Table 4 shows that gestational week, gestational week squared, season, total energy intake, dietary intake of vitamin D, and multivitamin supplementation over the previous 14 days were related to the 25(OH)D levels in the study population.

**Table 4:** Results of the multivariate linear mixed model analyses of factors related to plasma concentrations of vitamin D in women during pregnancy and after birth.

Factors	β	SE	*p*
Gestational week/weeks after birth	0.484	0.115	<0.001
Gestational week squared	-0.007	0.001	<0.001
Season^1^	-12.4	1.12	<0.001
Total energy intake^2^	-0.004	0.002	0.089
Vitamin D (dietary intake)^2^	1.63	0.53	0.002
Multivitamin the latest 14 days	-5.2	1.7	0.002

^1^Summer: April 15 –September 15; Winter: September 16 –April 14 [11].

^2^ Energy and nutrient intake increased by 25% to adjust for underreporting resulting from shortening of the FFQ by 25% from the original validated version [17].

**Fig 2:**
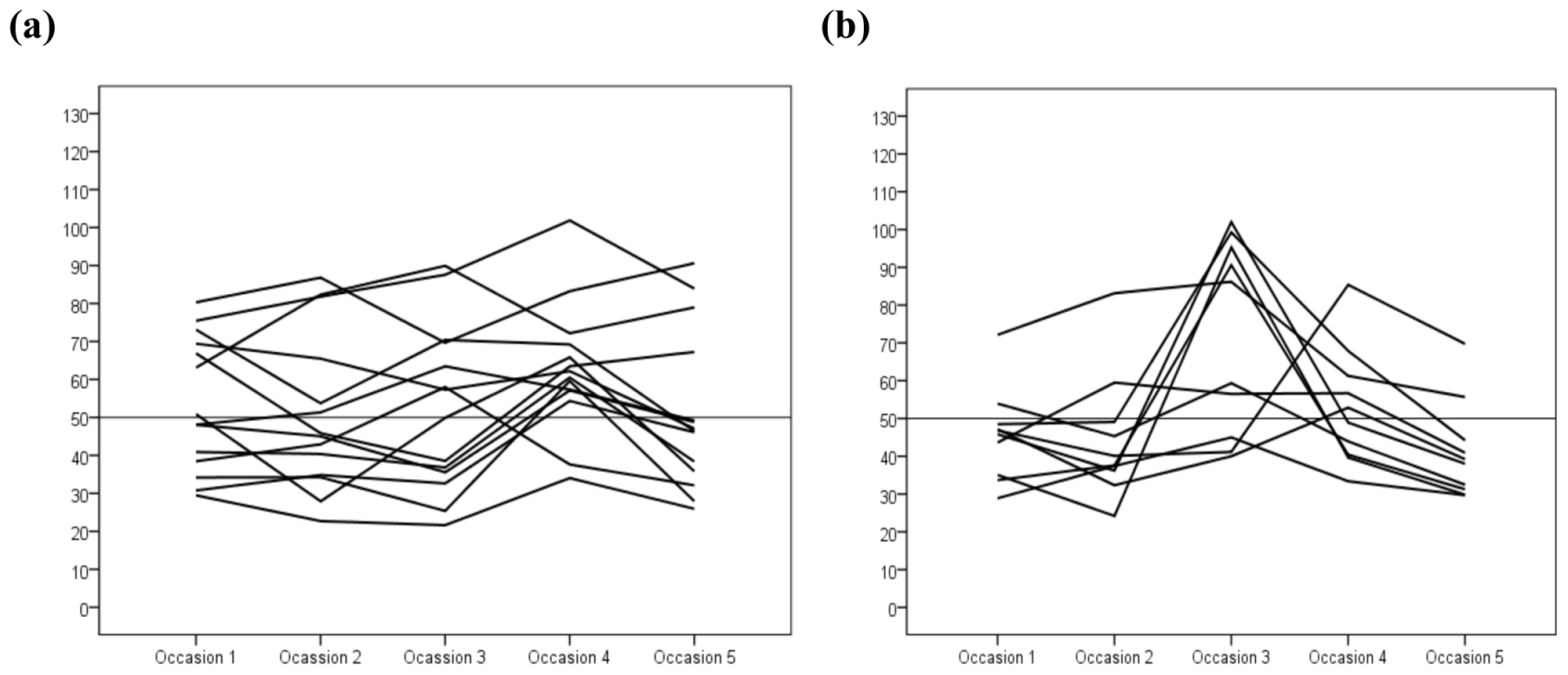
Vitamin D levels (nmol/L) in pregnant women divided by the month of first blood sampling. Each line represents one women and her vitamin D levels at 12 weeks after conception (occasion 1), at gestational weeks 21 (occasion 2) and 35 (occasion 3), and at 12 (occasion 4) and 29 (occasion 5) weeks post-partum. The mean gestational length was 39.4 weeks.

In total, 126 women attended all five sampling sessions. Of these women, 24 had their first sampling occasion in November (n = 14, Fig 2a) or January (n = 10, Fig 2b). Thus their individual longitudinal patterns of plasma 25(OH)D levels are shown in Fig 2. Individual longitudinal patterns for all 126 women divided by the month of the first sampling occasion are shown in S1 and S2 Figs.

It is notable that the seasonal variation influenced the baseline values and longitudinal patterns of vitamin D levels during and after pregnancy. Women entering pregnancy in August had more stationary levels than women entering pregnancy in January, when the vitamin D levels followed a peak-shaped pattern. This more stationary pattern was observed in women entering pregnancy during April–September, and the more peak-shaped pattern was observed in those entering pregnancy during October–March (S1 and S2 Figs).

## Discussion

The main findings of the present study were that the plasma vitamin D levels increased with gestational length, with the highest levels observed in late pregnancy. Season, supplementation, and vitamin D intake from food were significantly associated with plasma vitamin D levels. In total, at least one-third of the women had insufficient plasma levels of vitamin D (<50 nmol/L) during both pregnancy and the first 7 months after giving birth. This result suggests that, for a significant portion of woman living in the sub-arctic area of Sweden, sun exposure and intake from foods do not provide sufficient vitamin D. Because the present data indicate that the recommendations for pregnant women in the Nordic Nutrition Recommendations (NNR) are not met, supplementation may be considered [12].

The finding of increasing vitamin D levels during pregnancy is in accordance with several other, but not all, longitudinal studies [13,19]. For example, Møller et al [20] found higher levels in the 2^nd^ trimester and lower levels in the 3^rd^; however, this pattern was also seen in a parallel control group, and the conclusion was that pregnancy did not affect vitamin D levels *per se*. Zhang et al [21] found decreasing levels during pregnancy in a longitudinal study in Irish women, and the time course did not differ between women recruited from May-July and those recruited from August-September. The reason for these seemingly conflicting results between different studies is not understood, but several factors may not be comparable between the different studies, including fasting conditions, method of vitamin D analysis, latitude, exposure to UV light, lifestyle habits, and genetic polymorphisms in the vitamin D receptor and the vitamin D-binding protein.

Summer season and dietary and supplement intake were correlated with higher plasma levels of vitamin D, in accordance with most studies in other settings [14,22,23]. Among the pregnant women in this study, season had the strongest effect on the individual profile of vitamin D in plasma, i.e., the time of year at which pregnancy began and the time of the year at which follow-up samples were collected. Thus, when the pregnancy started in October to January, the peak vitamin D levels in plasma in gestational week 35 occurred during the summer season. This profile coincides with the need for calcium for tissue mineralization in the fetus [3]. Thus, the fetus accumulates approximately 30 g of calcium before birth, with maximal absorption in late pregnancy. Furthermore, during lactation, approximately 200 mg/day of calcium is added via breast milk [24]. When pregnancy starts in May-December, the opposite pattern occurs, i.e., vitamin D levels decline during pregnancy and rise in the following summer season after birth. To prevent any health risk to the mother and child, adequate levels of vitamin D should be maintained, especially during late pregnancy and the first two years of life. To ensure the intake of adequate levels, counseling on adequate intake from foods and eventually also supplementation should be considered at least in the winter in pregnant women living in northern latitudes [12]. However, the ground should be further evaluated in prospective interventional studies before it is applied as a general message to the population.

Our findings of insufficient levels (<50 nmol/L) of vitamin D in at least one-third of pregnant women and a low average intake of vitamin D (5 μg/day) are similar to those of other studies worldwide [19,20,25]. The intake we have reported from foods is half of the recommended amount according to the NNR [12]. Vitamin D is found mostly in fatty fish varieties, a few plants, eggs, liver, some mushrooms, and fortified dairy products. However, dietary recommendations for fish intake during pregnancy vary between countries. According to the National Food Agency in Sweden, some restrictions are given for fish from the Baltic sea due to toxins [26]. The intake of fish containing vitamin D, such as wild salmon, herring, walleye, perch, trout, halibut, mackerel, sardines, and tuna fish, is recommended once to twice a week, but care should be paid to the type and origin of the fish. According to a focus group study by Wennberg et al [27], pregnant women reported researching dietary information themselves after failing to obtain good advice from their midwife, and they sometimes found it difficult to keep track of fish-related information and avoid certain fish.

Dietary restrictions may be perceived as inhibitory and also worrying, particularly when some foods may be dangerous to the health of the child. Counseling should therefore be directed and specific to pregnant women. Although existing studies show conflicting results regarding the relationship between low levels of vitamin D and adverse pregnancy outcomes [25,28], Harvey et al [29] concluded that current knowledge is not sufficient to support definite recommendations for supplements during pregnancy. Low vitamin D levels are a risk factor for the health of the mother and optimal absorption of calcium to the fetus that should be acknowledged and addressed.

To our knowledge, this study is the first in Sweden to demonstrate intraindividual variation in vitamin D levels during pregnancy and postpartum in women living in the sub-Arctic region of the country. The strengths of the study are the repeated sampling from early pregnancy to 7 months after birth, the strictly standardized regimens that were used for sampling and information retrieval, and the recording of food intake, including supplement usage, in the same women at all visits. This design made it possible to follow each woman individually for approximately 1.5 years and monitor changes in dietary intake and plasma vitamin D levels. Another strength is that the study group was ethnically homogenous, i.e., only 13 of the 184 pregnant women in the study were of non-Swedish origin. This strength limits bias due to skin pigmentation and clothing habits. The use of LC-MS/MS for vitamin D analysis, which allows detection of both the D3 and D2 forms for total 25(OH)D estimation, is also a strength. The limitations include lack of data on individual sunlight exposure and travel to southern latitudes during the winter season, which would have likely improved the explanatory power of the multivariate model of factors affecting vitamin D levels. A further limitation is that the FFQ is not particularly designed to record vitamin D intake, but rather to monitor overall dietary intake. Additionally, the use of an FFQ is known to be biased by imprecise recording of unhealthy foods, often leading to an underestimation of energy and nutrients [30]. Aside from the effect of the comparably short FFQ used in the present study on total estimated energy and nutrient intake, obesity is the factor most strongly associated with bias in diet recordings [31]. In the present study, 13 women had a BMI ≥30 (obese), but inclusion of BMI as a covariate did not affect modeling outcome and therefore was not included in the final model. We are well aware of that several t-tests were performed in this study, which could increase the probability of getting false significances. Therefore significant outcomes should be interpreted with care. These limitations should be kept in mind before the results of the study are transferred to women living under different conditions and with different genetic profiles.

The clinical implications of the present study are that women living at the most northern latitudes who are pregnant during the winter should be a target for attention in antenatal care. Dietary counseling should be individualized and include at-risk individuals and toxic aspects of foods in the region. An unbalanced diet and low intake of vitamin D from food should be addressed with greater effort by promoting an increased intake of foods containing vitamin D and likely the use of supplements. Pregnancy is a suitable time to advocate a healthy lifestyle to minimize the risk of non-communicable diseases in the child (and family members) as the parents-to-be are usually concerned about providing the child with the best possible environment in the uterus and in infancy [18].

## Supporting Information

S1 Fig a-f. Individual longitudinal pattern of Vitamin D levels (nmol/L) divided by the month of the first blood sampling. Each line represents one women and her vitamin D levels at 12 weeks after conception (occasion 1), at gestational weeks 21 (occasion 2) and 35 (occasion 3), and at 12 (occasion 4) and 29 (occasion 5) weeks post-partum. The mean gestational length was 39.4 weeks. Figures labeled a-f represent women with sampling start in January, February, March, April, May and June, respectively.
(TIF)

S2 Fig g-l. Individual longitudinal pattern of Vitamin D levels (nmol/L) divided by the month of first blood sampling. Each line represents one women and her vitamin D levels at 12 weeks after conception (occasion 1), at gestational weeks 21 (occasion 2) and 35 (occasion 3), and at 12 (occasion 4) and 29 (occasion 5) weeks post-partum. The mean gestational length was 39.4 weeks. Figures labeled g-l represent women with sampling start in July August, September, October, November and December, respectively.
(TIF)

## Abbreviations

25(OH)D: 25-hydroxyvitamin D
LC-MS/MS: Liquid chromatography-tandem mass spectrometry
FFQ: Food Frequency Questionnaire
