# Supplementary figures and images for: Vitamin D Status during Pregnancy: A Longitudinal Study in Swedish Women from Early Pregnancy to Seven Months Postpartum
Source: PLoS One. 2016 Mar 3;11(3):e0150385. doi: 10.1371/journal.pone.0150385 (PMC4777524; doi:10.1371/journal.pone.0150385)

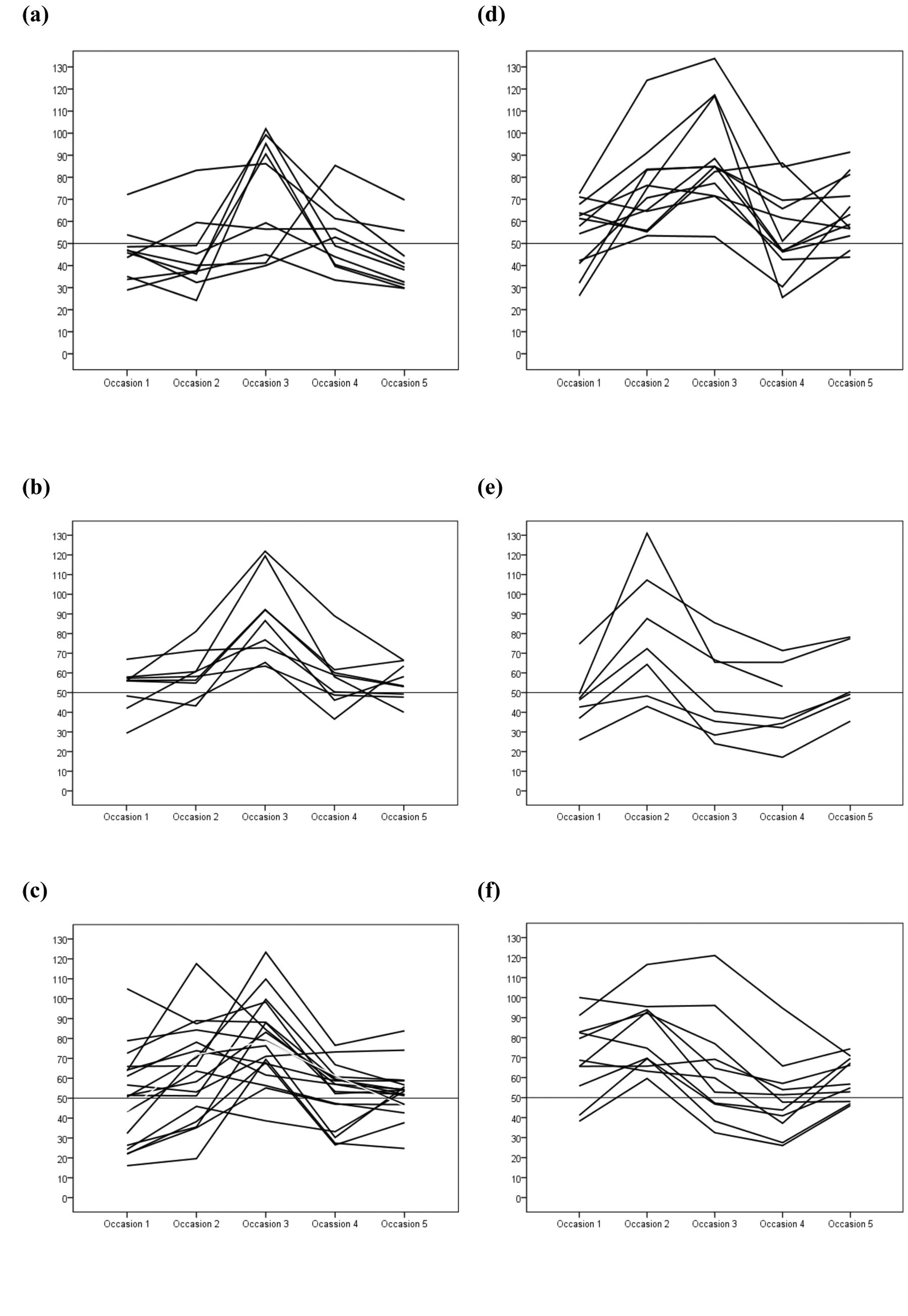

Supplement: S1 Fig — Each line represents one women and her vitamin D levels at 12 weeks after conception (occasion 1), at gestational weeks 21 (occasion 2) and 35 (occasion 3), and at 12 (occasion 4) and 29 (occasion 5) weeks post-partum. The mean gestational length was 39.4 weeks. Figures labeled a-f represent women with sampling start in January, February, March, April, May and June, respectively. (TIF) [file pone.0150385.s001.tif]

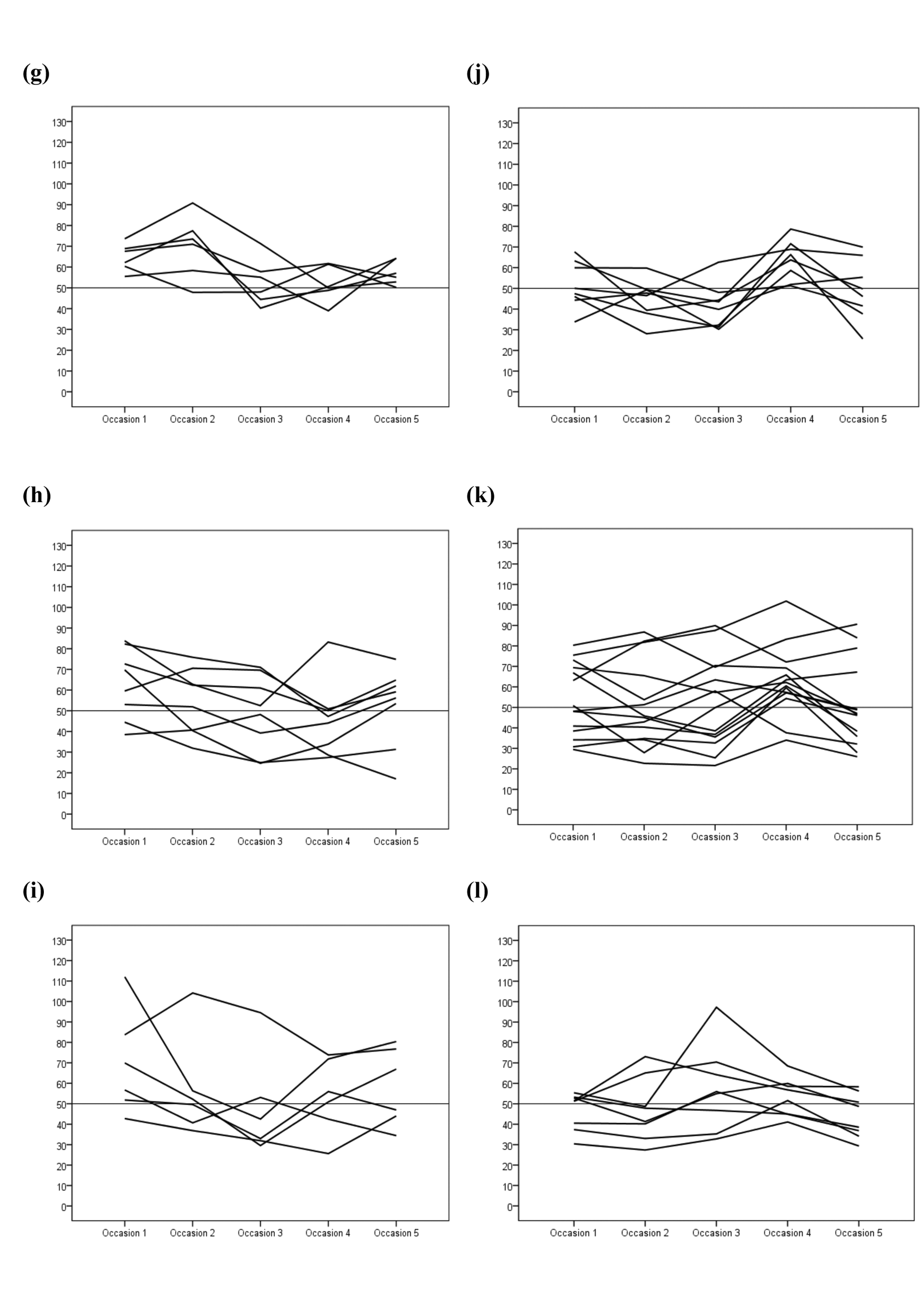

Supplement: S2 Fig — Each line represents one women and her vitamin D levels at 12 weeks after conception (occasion 1), at gestational weeks 21 (occasion 2) and 35 (occasion 3), and at 12 (occasion 4) and 29 (occasion 5) weeks post-partum. The mean gestational length was 39.4 weeks. Figures labeled g-l represent women with sampling start in July August, September, October, November and December, respectively. (TIF) [file pone.0150385.s002.tif]
